# Regulation of Polycomb Repression by *O*-GlcNAcylation: Linking Nutrition to Epigenetic Reprogramming in Embryonic Development and Cancer

**DOI:** 10.3389/fendo.2019.00117

**Published:** 2019-02-27

**Authors:** Amélie Decourcelle, Dominique Leprince, Vanessa Dehennaut

**Affiliations:** Université de Lille, CNRS, Institut Pasteur de Lille, UMR8161, M3T: Mechanisms of Tumorigenesis and Targeted Therapies, Lille, France

**Keywords:** O-GlcNAcylation, Polycomb, epigenetic, drosophila development, cancer

## Abstract

Epigenetic modifications are major actors of early embryogenesis and carcinogenesis and are sensitive to nutritional environment. In recent years, the nutritional sensor *O*-GlcNAcylation has been recognized as a key regulator of chromatin remodeling. In this review, we summarize and discuss recent clues that OGT and *O*-GlcNAcylation intimately regulate the functions of the Polycomb group proteins at different levels especially during *Drosophila melanogaster* embryonic development and in human cancer cell lines. These observations define an additional connection between nutrition and epigenetic reprogramming associated to embryonic development and cancer.

## Introduction

Epigenetics refer to inherited changes in gene expression that do not involve changes in the underlying DNA sequence (a change in phenotype without a change in genotype). Actually, epigenetic modifications such as DNA methylation and histones post-translational modifications drive reading of the genes through the modulation of chromatin topology that governs DNA accessibility to transcriptional machinery. Epigenetic changes are a natural phenomenon: crucial epigenetic reprogramming events occur during germ cell development and early embryogenesis (1–3). Furthermore, the levels and turnover of epigenetic marks can be influenced directly and indirectly by several factors, including lifestyle and environment, which can lead to a modification of gene expression patterns and, consequently, affect our health for better or worse (4). In that sense, several studies have highlighted the key role of diet and nutritional compounds in the epigenetic regulation of gene expression, especially in the physiopathology of cancers. For instance, it was shown that resveratrol and grape seed proanthocyanidins found in red wine induced a decrease of cell viability and colony forming ability in two human breast cancer cell lines, MDA-MB-231 and MCF-7, with a decrease of histone deacetylase (HDAC) and DNA methyl transferase (DNMT) activities (5). Another study has demonstrated that EGCG (EppiGalloCatechin Gallate), a polyphenolic compound found in green tea, had the ability to inhibit Acute Promyelocytic Leukemia (APL) cells proliferation and to induce apoptosis by downregulating epigenetic modifiers such as DNMT1, HDAC1, HDAC2, the histone methyl transferase (HMT) G9a and some components of the Polycomb Repressive Complex 2 (PRC2) (6). These studies, among others, proved that food consumption can influence epigenetic modifications by directly affecting activities of “writers” and “erasers” of epigenetic modifications and have contributed to the emergence of the concept of “epigenetic diet” which may have anti-cancer properties (7). On the contrary, many other studies support the hypothesis of a close link between nutritional disorders (obesity, metabolic syndrome, type 2 diabetes …), well-known risk factors for many cancers, and epigenetic reprogramming linked to carcinogenesis (8). For example, hyperglycemia has been shown to increase the chemoresistance of several prostate cancer cell lines to chemotherapy through the increase of histone H3 and H4 acetylation on the promoter region of insulin like growth factor binding protein 2 (IGFB2), a key player of prostate cancer progression (9). Wu et al. recently reported a decrease in DNA cytosine hydroxymethylation (5hmC, the first step of DNA demethylation catalyzed by the ten-eleven translocation (TET) family of dioxygenases) in peripheral blood mononuclear cells (PBMCs) derived from diabetic individuals in comparison with healthy one (10). They demonstrated that this reduction of 5hmC was the result of glucose-regulated phosphorylation of TET2 by the nutrient and energy sensor AMPK (AMP-activated kinase) and that elevated glucose levels interfere with the expression of numerous cancer-associated genes in a TET2-dependent manner. Among the elements that could also connect nutrition to epigenetic reprogramming related to development and cancer, the nutritional sensor *O*-linked-β-N-acetylglucosaminylation (*O*-GlcNAcylation) has emerged, during the last decade as a key epigenetic regulator of gene expression (11, 12).

## *O*-GlcNAcylation: A Nutritional Sensor That Regulates Chromatin Remodeling

**Figure 1:**
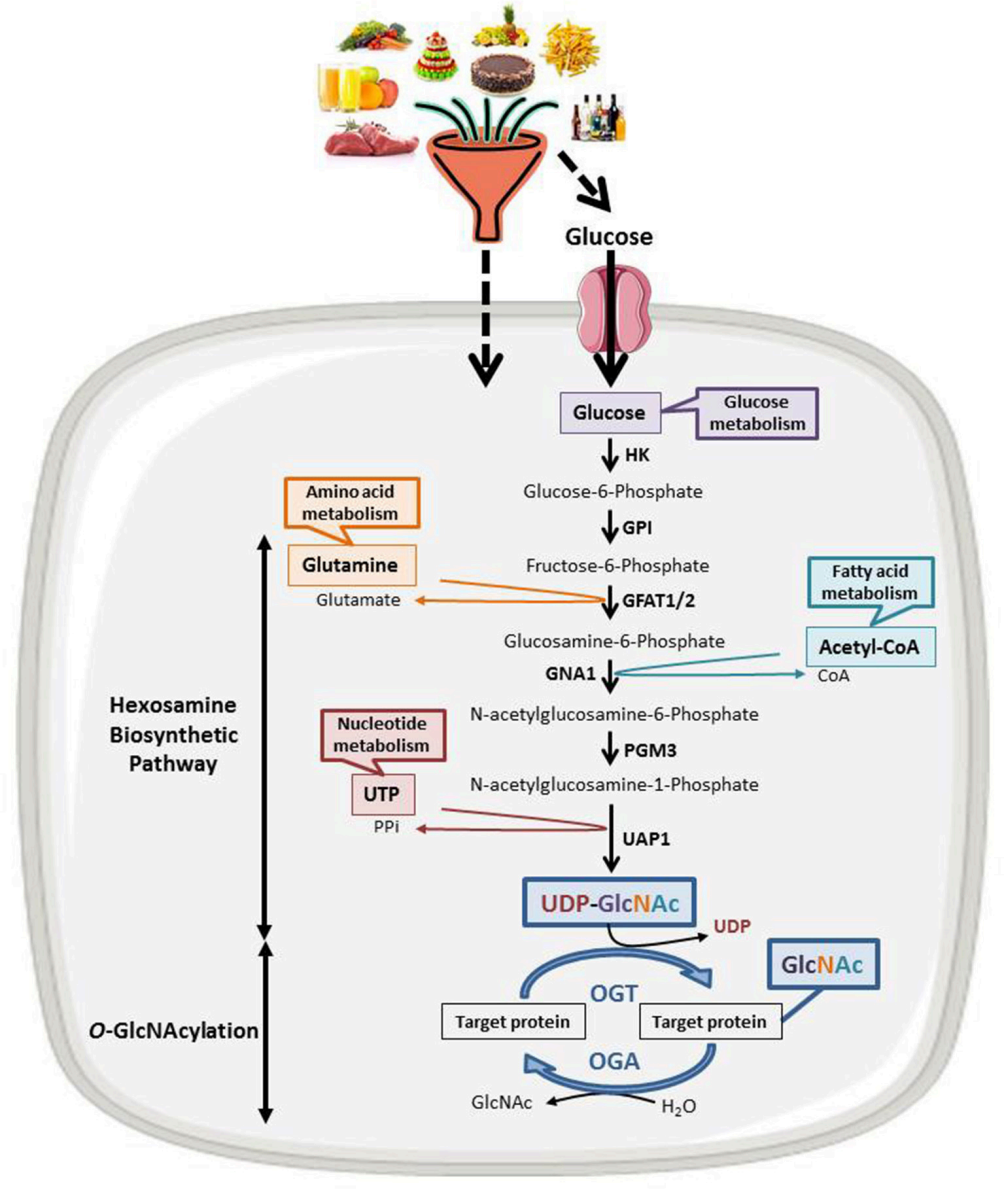
The nutritional sensing *O*-GlcNAcylation. UDP-GlcNAc, OGT's nucleotide sugar donor, is provided by the Hexosamine Biosynthetic Pathway (HBP) at the crossroad of glucose, amino acids, fatty acids and nucleotides metabolisms. UDP-GlcNAc levels are thus tightly correlated to the nutritional status of the organism. *O*-GlcNAcylation levels are regulated by a unique couple of enzymes: OGT that catalyzes the transfer of GlcNAc from UDP-GlcNAc onto the target protein and OGA that hydrolyzes the residue. HK, hexokinase; GPI, glucose-6-phosphate isomerase; GFAT, fructose-6-phosphate amidotransferase; GNA1, Glucosamine 6-phosphate N-acetyltransferase; PGM3, phosphoacetylglucosamine mutase; UAP1, UDP-N-acetylhexosamine pyrophosphorylase.

Discovered in 1984 (13), *O*-GlcNAcylation is a reversible post-translational modification of cytosolic, nuclear, and mitochondrial proteins that consists in the covalent linkage of a unique residue of N-acetylglucosamine (GlcNAc) to serine and threonine moieties of target proteins (Figure 1). This post-translational modification is an important actor of cell signaling. *O*-GlcNAcylation targets and regulates thousands of proteins by monitoring their expression, stability, interaction with partners and subcellular localization (14). *O*-GlcNAcylation levels are regulated by a unique couple of enzymes: OGT (*O*-GlcNAc Transferase) catalyzes the transfer of GlcNAc from UDP-GlcNAc onto targeted proteins and OGA (*O*-GlcNAcase) hydrolyzes the residue (15). OGT activity and thus *O*-GlcNAcylation levels are closely dependent upon the concentration of the nucleotide sugar donor UDP-GlcNAc. UDP-GlcNAc is synthetized through the hexosamine biosynthetic pathway (HBP) at the crossroad of carbohydrates, amino-acids, fatty acids, and nucleotides metabolisms (Figure 1) and thus concentration of UDP-GlcNAc in cells is highly responsive to nutrients flux. Consistently, cell culture in high concentration of glucose or glucosamine is sufficient to increase *O*-GlcNAcylation levels in numerous cancer cell lines including HT29, HCT116, MCF7 and HepG2 (16–18). *In vivo*, increased *O*-GlcNAcylation has also been evidenced in the colon and the liver of mice refed or force-fed with glucose or glucosamine in comparison with fasting mice (18–20), in mice fed with a high carbohydrate diet and in obese mice (*ob/ob*) compared, respectively, to the normal diet and the wild-type mice (21, 22). Taken together, these studies, amongst others, sustain the hypothesis that UDP-GlcNAc and *O*-GlcNAcylation can be considered as nutritional sensors that can relay the effects of excessive food supply, obesity, and any other metabolic disorders associated to increased risk of cancers. Many studies have also clearly shown that a disruption in *O*-GlcNAcylation homeostasis is found in many cancers; moreover, aberrant *O*-GlcNAcylation contributes to the etiology of cancers at several levels. The role of *O*-GlcNAcylation in cancer emergence and progression has been extensively reviewed (23, 24). In recent years, *O*-GlcNAcylation has emerged as a novel epigenetic modifier affecting chromatin remodeling and gene expression. In that respect, *O*-GlcNAcylation is itself part of the histone code and regulates the occurrence of other PTMs defining this code and more particularly methylation by modulating the function of several methyltransferases like CARM1 and MLL5. OGT interacts in a complex interplay with the DNA demethylase TET family and regulates the activity of several co-repressor complexes among which NuRD and mSin3A. These different aspects of the role of *O*-GlcNAcylation in the epigenetic regulation of gene expression has been reviewed elsewhere (11, 12, 25, 26) and will not be discussed here.

This mini-review will summarize and discuss recent data focusing on how OGT and *O*-GlcNAcylation influence directly and indirectly the function of the Polycomb group (PcG) proteins, master regulators of embryogenesis and actors of human carcinogenesis.

## *O*-GlcNAcylation Regulates PcG Proteins Functions During *Drosophila Melanogaster* Development

### Organization of the PcG Proteins in *Drosophila Melanogaster*

**Figure 2:**
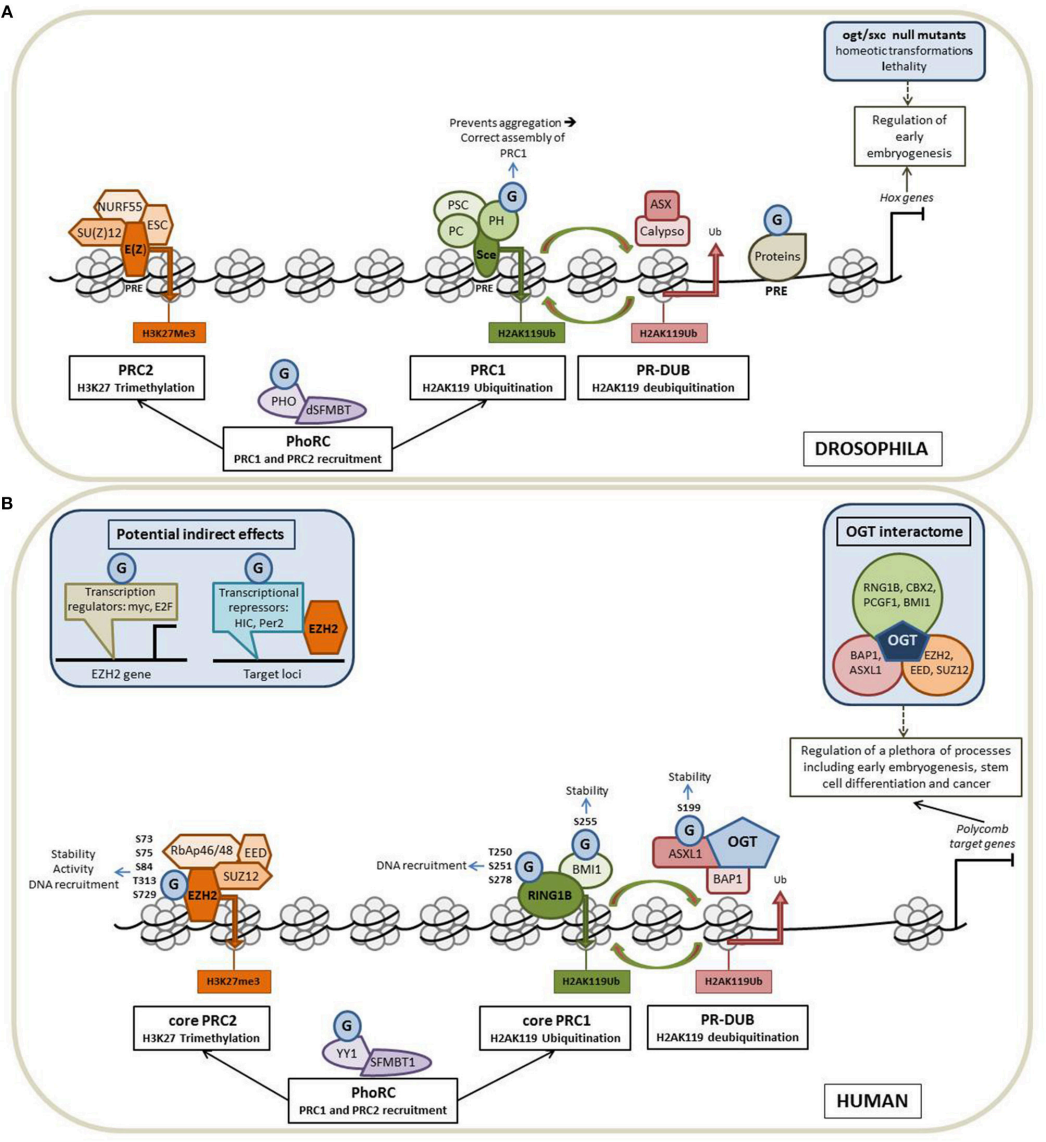
*O*-GlcNAcylation intimately regulates the Polycomb proteins functions. Schematic representation of the organization of the PcG proteins in Drosophila **(A)** and in Human **(B)** and of how *O*-GlcNAcylation processes contribute to the regulation of the Polycomb-mediated gene repression in the two models. **(A)** In fly, PcG proteins maintain the repression state of *Hox* genes whose expression patterns define the establishment of the antero-posterior axis of the embryo. PcG proteins form three broad groups of polycomb repressive complexes (PRCs): PRC1, PRC2 and Polycomb Repressive DeUBiquitinase (PR-DUB). Each PRC modifies and remodels chromatin by distinct mechanisms tuned by variable compositions of core and accessory subunits. The core PRC1 is composed of PH, PSC, PC, and Sce that catalyzes H2AK119 ubiquitination. This repressive mark can be removed by the PR-DUB complex (association of Calypso and ASX). NURF55, SU(Z)12, ESC, and E(Z) forms the PRC2 core complex in which E(Z) has a H3K27 methylation activity. The fourth complex, PhoRC (association of PHO and dSFMBT) helps the recruitment of PRC1 and PRC2 to their target genes. PcG proteins are recruited to their target genes through the recognition of well-characterized regulatory DNA sequences called PREs (Polycomb Responsive Elements). OGT is also a PcG protein encoded by the supersexcomb (*sxc*) gene and *O*-GlcNAcylation regulates the PcG mediated repression at several levels (see the text for more details). G: *O*-GlcNAcylation. **(B)** In Human, PcG proteins repress numerous genes regulating a plethora of cellular processes, including early embryogenesis, stem cell differentiation and cancer. As in fly, Human Polycomb proteins are organized into four main complexes. The core PRC1 is composed of RING1 proteins (RING1A or RING1B), which display E3 ubiquitin ligase activity and one of the six Polycomb group ring-finger domain proteins (PCGF1–PCGF6). The PR-DUB is composed of BAP1 and ASXL 1 or 2. The association of RbAp46/48, EED, SUZ12, and EZH2 leads to the formation of the PRC2 core complex. PhoRC includes YY1 and SFMBT1. As in fly, OGT interacts and modifies several Human PcG proteins to regulate their functions and *O*-GlcNAcylation could also play a more indirect role through the modification of factors regulating the expression of PcG (see the text for details).

The polycomb group (PcG) proteins form a diverse and conserved group of epigenetic modifiers and transcriptional regulators. PcG proteins were initially discovered in *Drosophila melanogaster* (27). In fly, PcG proteins maintain the repression state of *Hox* genes whose expression patterns define the establishment of the antero-posterior axis of the embryo. PcG proteins form three broad groups of polycomb repressive complexes (PRCs) known as PRC1, PRC2, and Polycomb Repressive DeUBiquitinase (PR-DUB). Each PRC modifies and remodels chromatin by distinct mechanisms tuned by variable compositions of core and accessory subunits (Figure 2A). The PRC1 is composed of Sce, PH, PSC and PC. Sce harbors a H2AK119 ubiquitination activity. This H2AK119Ub repressive mark can be removed by the PR-DUB complex which is composed of Calypso and ASX. The association of NURF55, SU(Z)12, ESC and E(Z) leads to the formation of the PRC2. E(Z) has a H3K27 methylation activity. A fourth complex, PhoRC, allows the recruitment of PRC1 and PRC2 to their target genes and includes PHO and dSFMBT (28).

### Fruit Fly OGT Is a Polycomb Protein Essential to Early Embryogenesis

Intriguingly, two independent studies published in 2009 showed that the fruit fly *O*-GlcNAc transferase was encoded by the *Super sex combs* (*sxc*) gene (29, 30) characterized for the first time 25 years earlier as a gene belonging to the Polycomb family (31). Therefore, with rapid generation time and genetic manipulation facilities, *Drosophila* has been a model of choice for understanding the role of *O*-GlcNAcylation in development. Several studies have highlighted homeotic transformations and lethality phenotypes of various null *sxc/ogt* mutants (29–34) and have been ascribed to the glycosyltransferase activity of drosophila OGT (33, 34) thus demonstrating the crucial role of *ogt/sxc* and *O*-GlcNAcylation processes in early development. Consistent with these results, in an elegant approach consisting of probing *O*-GlcNAcylated proteins by harnessing the *O*-GlcNAc binding properties of a catalytic mutant of a bacterial *O*-GlcNAcase (*Cp*OGA^D298N^), Mariappa et al. demonstrated that the *O*-GlcNAcome was dynamic during the time course of *Drosophila* embryonic development (35). This OGA mutant was then used as a trap to enrich *O*-GlcNAcylated proteins from Drosophila embryo lysates and allowed the identification of the *O*-GlcNAcome associated with Drosophila embryogenesis (36). The authors identified more than 2,000 putative *O*-GlcNAcylated proteins, the majority of which being chromatin-associated proteins that include the HDACs Rpd3 and HDAC3, the putative HAT Enok, the bromodomain containing homeotic protein female sterile [fs(1)h; Brd2 ortholog] and several transcription factors among which Dp, Taf6, Cand1, and fkh. The authors were also able to map several *O*-GlcNAcylation sites on 43 proteins whose half of which are involved in anatomical structure development and morphogenesis according to gene ontology enrichment analysis.

### Regulation of PcG Mediated Repression by O-GlcNAcylation

In Drosophila, PcG proteins are recruited to their target genes through the recognition of well-characterized regulatory DNA sequences called PREs (Polycomb Responsive Elements) (37). By ChIP-seq experiments performed in imaginal disc cells from Drosophila larvae, Gambetta and collaborators identified 1,138 sites occupied by *O*-GlcNAcylated proteins among which 490 co-localized with PREs suggesting that *O*-GlcNAcylation regulates the PcG mediated repression of *HOX* genes (29). Such a binding of *O*-GlcNAcylated proteins onto PREs has also been evidenced in two other independent studies (38, 39). This binding of *O*-GlcNAcylated proteins to PREs is in line with the results from Ingham showing that *ogt/sxc* is required for the selective repression of *HOX* genes in different larval segments (31). The Pho repressive complex (PhoRC) is involved in the recruitment of PRC1 and PRC2 (Figure 2A), thus the presence of Pho at genomic loci is often considered as a marker for identification of PREs. By ChIP-chip experiments conducted in S2 cells, Akan et al. demonstrated that *O*-GlcNAc and Pho co-occupied many chromatin regions including the *HOX* genes clusters thus reinforcing the hypothesis of the role of *O*-GlcNAcylation in PcG mediated repression (38). Nonetheless, *ogt/sxc* null mutants do not present either any binding modification of PHO and E(Z) to PREs or any difference in H3K27me3 in comparison with wild-type drosophila suggesting that *ogt/sxc* is not essential for PRC2 recruitment to target genes and H3K27me3 activity (29). However, in the same study, the authors demonstrated that PH (Polyhomeotic) is *O*-GlcNAcylated and observed a decrease of its binding on the majority of the PREs in *sxc/ogt* null mutants, underlying the role of *O*-GlcNAcylation in the interaction of PH with DNA. The same team demonstrated that *O*-GlcNAcylation prevents the self-aggregation of PH and is required for the correct assembly of PRC1 (32). Lastly, it was also demonstrated that PHO is *O*-GlcNAcylated but the role of the glycosylation on PHO properties has not been so far investigated (39). However, this post-translational modification does not seem to modify its DNA recruitment according to the results from Gambetta et al. (29).

## *O*-GlcNAcylation Regulates the Polycomb Proteins Functions in Human Cancer Cell Lines

### Organization of the Polycomb Group Proteins in Mammals

Numerous orthologs of Drosophila PcG proteins have been identified in mammals and revealed that the PcG system is much more complex in mammals than in fly (40) (Figure 2B). In mammals, PRC1 is subdivided into canonical (cPRC1) and non-canonical complexes (ncPRC1). These two PRC1 complexes share a core complex that is composed of RING1 proteins (RING1A or RING1B), which display E3 ubiquitin ligase activity mediating ubiquitylation of histone H2A on lysine 119 (H2AK119Ub), and one of the six Polycomb group ring-finger domain proteins (PCGF1–PCGF6). The repressive mark H2AK119Ub can be removed by the PR-DUB complex which is composed of BAP1 and ASXL. The association of RbAp46/48, EED, SUZ12 and EZH2 leads to the formation of the mammalian PRC2 core complex. The association of several accessory proteins with the core PRC2 complex defined two subtypes of PRC2: PRC2.1 and PRC2.2. PRC2.1 is defined by its mutually exclusive binding of one of the three Polycomb-like homologs (PCLs) PHF1, PHF19, or MTF2; PRC2.2 is defined by the presence of the zinc-finger proteins AEBP2 and JARID2, which enhance enzymatic activity of the complex and regulate its chromatin binding affinity. Finally, the PhoRC includes YY1 and SFMBT1, and allows the recruitment of PRC1 and PRC2 to their target genes (28, 41).

In mammals, PcG proteins repress *Hox* genes but also control the expression of numerous other genes regulating a plethora of cellular processes, including X chromosome inactivation, genomic imprinting, cell cycle control and stem cell biology (40). Furthermore, misexpression or mutation of many PcG components has been evidenced in several cancers. Many studies have demonstrated that PcG proteins can play an oncogenic role. For example, high levels of EZH2 and the H3K27me3 mark often correlate with a poor prognosis in prostate tumors (42). In colorectal cancer, elevated expression of EZH2, BMI1, and SUZ12 in correlation with advances stages of the disease and poor prognosis has also been evidenced (43). In line of this, the development of PcG-specific inhibitors, particularly compounds targeting EZH2, is an active area of investigation for the treatment of cancers (44) and the in-depth understanding of how PcG functions are regulated, such as by post-translational modifications, is a real challenge to improve the development of such therapeutic tools.

### Regulation of PcG Mediated Repression by O-GlcNAcylation

Recent data collected from proteomic analyses aiming at identifying the OGT interactome in Hela cells revealed that the glycosyltransferase interacts with lots of PcG proteins belonging to PRC1, PRC2, PR-DUB, and PhoRC complexes: EZH2, EED, SUZ12, RNF2 (also called RING1B), CBX2, PCGF1, BMI1, BAP1, and ASXL1, thus suggesting that *O*-GlcNAcylation is also a master regulator of PcG functions in mammals (45). Another recent study, dedicated to mapping the human Polycomb complexome, showed that OGT is also an accessory protein of the PR-DUB complex (H2AK119Ub eraser) reinforcing the involvement of *O*-GlcNAcylation in the regulation of PcG (46). In the same way, ChIP Seq experiments performed in the colon cancer cell line HT29 demonstrated that *O*-GlcNAcylated proteins and H3K27 trimethylation were found together at the promoter region of 61 genes among which *MYBL* whose epigenetic regulation by *O*-GlcNAcylation affects the population of cancer stem cells (47). ChIP assays performed in the breast cancer cell line MCF7 indicated that the promoter regions of 16 potential tumor suppressor genes were bound by OGT and were enriched in EZH2 and H3K27me3 in an OGT-dependent manner (48), also arguing for a role of *O*-GlcNAcylation in the regulation of the PRC2-mediated repression in the context of cancer. Interestingly, recent data from Jiang et al. revealed that OGT and EZH2 expression were both post-transcriptionally repressed by microRNA-101 (49). The authors also showed that miRNA-101 was epigenetically silenced by OGT and EZH2 in several colorectal cancer cell lines resulting in the upregulation of the two enzymes in metastatic colorectal cancer in a vicious cycle fashion.

### OGT Interacts With and Modifies Several PcG Proteins

To date, among the OGT-interacting PcG proteins, five have been demonstrated to be *O*-GlcNAcylated in Human: EZH2, BMI1 (also called PCGF4), RING1B, ASXL1, and YY1. *O*-GlcNAcylation of EZH2 was first evidenced in breast cancer MCF7 cells (48). In this study, the authors identified the serine 75 of EZH2 as the major *O*-GlcNAc site regulating the stability of the enzyme. Very recently, the same team performed further mass spectrometry analysis of EZH2-FLAG overexpressed in HEK 293T cells and identified four additional *O*-GlcNAcylation sites: S73, S84, T313, and S729 (50). By analyzing *O*-GlcNAcylation site mutants, the authors concluded that *O*-GlcNAcylation in the N-terminal region of EZH2 stabilizes the enzyme whereas the *O*-GlcNAcylation at S279 in the catalytic domain is essential for its methyltransferase activity. However, the role of *O*-GlcNAc modification on EZH2 is not so clear. While some studies confirm the role of *O*-GlcNAcylation in the regulation of EZH2 stability and catalytic activity (49, 51), others propose that the glycosylation regulates rather EZH2 recruitment to some of its target genes such as *FOXC1* (52). BMI1 was found to be *O*-GlcNAcylated at serine 255 in prostate cancer cells (53). *O*-GlcNAcylation of BMI1 prevents its proteasomal degradation and promotes its oncogenic activity. *O*-GlcNAcylation of RING1B was evidenced in human embryonic stem cells and mapped at residues threonine 250, serine 251 and serine 278 (54). By ChiP Seq experiments, the authors demonstrated that the non-GlcNAcylated form of RING1B preferentially binds to genes related to metabolism and cell cycle processes whereas *O*-GlcNAcylated-RING1B was found to the promoter region of genes related to neuronal differentiation. This means that *O*-GlcNAcylation might regulate RING1B DNA recruitment and targeting of the PRC1 complex to specific loci. It has been recently shown that OGT interacts also with ASXL1, a PR-DUB component, and drives its *O*-GlcNAcylation at serine 199 to regulate its stability (51). Finally the *O*-GlcNAcylation of YY1 has been demonstrated in muscle cells but its potential influence on the recruitment of PRC1 and PRC2 has not been yet investigated (55).

### Potential Indirect Regulation of PcG Functions by O-GlcNAcylation

Beyond the direct regulation of PcG functions by their own *O*-GlcNAcylation, the glycosylation could play a more distant role through the modification of factors regulating the expression of PcG even if it has not been demonstrated to date. For example, two transcriptional regulators of EZH2: c-myc (56) and E2F (57) are *O*-GlcNAcylated (58, 59). Although the mode of recruitment of the Polycomb proteins to their target genes is well-known in *Drosophila*, this mechanism is not clear in humans and remains to be clarified. Nevertheless, it involves several partners that direct the PcG proteins to specific loci. For example, the tumor suppressor gene HIC1 (60) and the co-repressor PER2 (61) allow the recruitment of PRC2 to some target genes. In the same vein, RING1B and EZH2 have been identified as Snail-interacting proteins (62, 63). Interestingly, these three PRC partners are *O*-GlcNAcylated (64–66); it can be therefore hypothesized, although not yet studied, that *O*-GlcNAcylation influences the interaction of the PRC complexes with these proteins.

## Conclusion

*O*-GlcNAcylation processes and epigenetic modifications are both sensitive to nutritional environment and have been evidenced as key regulators of embryogenesis and carcinogenesis. In this review, we summarized evidences that OGT and *O*-GlcNAcylation intimately regulate the functions of the Polycomb group proteins at different levels especially during *Drosophila melanogaster* embryonic development and in human cancer cell lines (Figure 2). Although, further works are required to clarify the roles of *O*-GlcNAcylation in PcG mediated repression, especially during cancer emergence and progression, all the data collected here sustain the hypothesis that *O*-GlcNAcylation is a new link between nutrition and epigenetic reprogramming of cancer cells. These observations could explain in part why nutritional disorders like diabetes or metabolic syndromes are often associated with the risk of cancer. Indeed, one can easily hypothesize that nutritional disorders, by increasing cellular levels of UDP-GlcNAc and *O*-GlcNAcylation leads to aberrant activity of PcG proteins misregulating genes driving carcinogenesis. Thus, in the next future, the *O*-GlcNAcylated forms of PcG proteins may be envisaged as diagnostic or prognostic tools in cancer and their targeting may also be studied as new therapeutic approaches.

## Author Contributions

AD and VD conceived the plan and wrote the review. DL revised it critically for important intellectual content.

### Conflict of Interest Statement

The authors declare that the research was conducted in the absence of any commercial or financial relationships that could be construed as a potential conflict of interest.

## Abbreviations

ASX: Additional Sex Combs
ASXL: Additional Sex Combs Like
BAP1: BRCA1 associated protein-1
BMI1 (PCGF4): B Lymphoma Mo-MLV insertion region-1
CARM1: Coactivator-associated Arginine Methyltransferase 1
CBX2: Chromobox Homolog 2
DNMT: DNA Methyl Transferase
EED: Embryonic Ectoderm Development
ESC: Extra Sex Combs
E(Z): Enhancer of Zeste
EZH2: Enhancer of Zeste Homolog 2
HAT: Histone Acetyl Transferase
HBP: Hexosamine Biosynthetic Pathway
HCF1: Host Cell Factor 1
HDAC: Histone Deacetylase
HIC1: Hypermethylated In Cancer 1
MLL5: Mixed Lineage Leukemia 5
NuRD: Nucleosome Remodeling and Deacetylase
NURF55: Nucleosome Remodeling Factor 55
OGA: *O*-GlcNAcase
*O*-GlcNAcylation: *O*-linked β-N-acetylglucosaminylation
OGT: *O*-GlcNAc Transferase
PC: Polycomb
PcG: Polycomb
PCGF: Polycomb group ring fingers
PER2: Period Circadian Regulator 2
PH: Polyhomeotic
PHO: Pleiohomeotic
PhoRC: Pho-Repressive Complex
PRC1: Polycomb Repressive Complex 1
PRC2: Polycomb Repressive Complex 2
PR-DUB: Pc-Repressive deubiquitinase
PRE: Polycomb Response Element
PSC: Posterior Sex Combs
RbAp46/48: Retinoblastoma Associated protein 46/48
RING1A/B: RING Finger Protein 1A/B
RNF2 (RING1B): Ring Finger Protein 2
Sce: Sex Comb Extra
SU(Z)12: Suppressor of Zeste 12
sxc: Super sex combs
TET: Ten Eleven Translocation
UDP-GlcNAc: Uridine DiPhosphate N-acetylglucosamine
YY1: Yin Yang 1.
